# LiNbO_3_ and LiTaO_3_ Coating Effects on the Interface of the LiCoO_2_ Cathode: A DFT Study of Li-Ion Transport

**DOI:** 10.1021/acsami.4c05737

**Published:** 2024-08-05

**Authors:** Zizhen Zhou, Huu Duc Luong, Bo Gao, Toshiyuki Momma, Yoshitaka Tateyama

**Affiliations:** † Graduate School of Advanced Science and Engineering, Waseda University, Shinjuku-ku, Tokyo 169-8555, Japan; ‡ Research Center for Energy and Environmental Materials (GREEN), National Institute for Materials Science (NIMS), Tsukuba, Ibaraki 305-0044, Japan; § Laboratory for Chemistry and Life Science, Institute of Innovative Research, Tokyo Institute of Technology, Midori-ku, Yokohama 226-8501, Japan; ∥ College of Materials Science and Engineering, Jilin University, Changchun, Jilin 130012, China

**Keywords:** solid-state battery, first-principles calculation, interface degradation, coating material, Li-ion migration

## Abstract

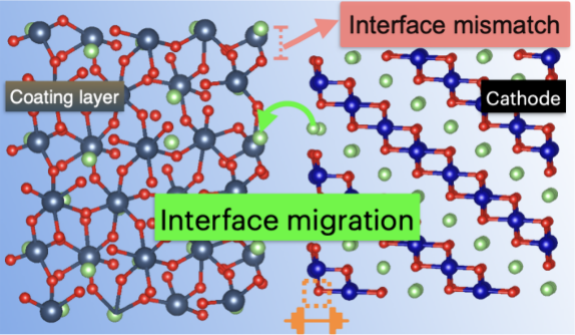

In solid-state batteries, the interface between cathodes and solid electrolytes is crucial and coating layers play a vital role. LiNbO_3_ has been known as a promising coating material, whereas recent studies showed its degradation via releasing oxygen and lithium during cycling. This computational study addresses the elucidation of essential characteristics of the coating materials by examining LiNbO_3_ and its counterpart LiTaO_3_ interfaces to a representative layered cathode, LiCoO_2_. Employing the interface CALYPSO method, we constructed explicit models of both coatings on LiCoO_2_. Our findings indicate that LiTaO_3_ offers easier Li^+^ migration at the interface due to the smaller difference in Li adiabatic potential at the interface, whereas LiNbO_3_ more effectively suppresses oxygen activity at high delithiation states via lowering the O 2p states. This comparative analysis provides essential insights into optimizing coating materials for improved battery performance.

## Introduction

In the rapidly evolving field of energy storage technology, solid-state batteries (SSBs) with solid electrolytes have emerged as a potential successor to traditional lithium-ion batteries (LIBs).^1,2^ These innovative systems promise enhanced safety, broader operational temperature ranges, and higher energy densities. Among various types of solid electrolytes, thiophosphate solid electrolytes (SEs), such as Li_10_GeP_2_S_12_ (LGPS) and argyrodites Li_6_PS_5_X (where X = Cl, Br, I), are emerging as frontrunners due to their high Li^+^ conductivities (10^–3^–10^–2^ S cm^–1^) and low-temperature processability.^3,4^ However, undesirable interface reactions between the solid electrolyte and the cathode material bring up challenges for the advancement of SSBs.^5^

Interface reactions in SSBs primarily manifest through two representative side reactions: the formation of a space-charge layer (SCL)^6−8^ and the mutual diffusion of elements.^9−12^ The SCL formation alters the local electrostatic environment at the interface, leading to interface resistance. Similarly, the mutual diffusion of elements results in compositional changes at the interface, further exacerbating the degradation process. Together, these phenomena contribute to a significant increase in interfacial charge transfer resistance, ultimately leading to poor battery performance.

To tackle these challenges, the deployment of coating materials at the cathode/electrolyte interface has been explored as a viable strategy to enhance interface stability and mitigate degradation, as well as suppress the interface resistance. LiNbO_3_ (LiNbO), in particular, has garnered attention for its effectiveness in suppressing adverse reactions at the interface, thus improving the structural integrity and efficiency of SSBs.^7^ The favorable properties of LiNbO, including its low electrical conductivity, robustness, and ability to facilitate Li^+^ transport (10^–5^–10^–6^ S cm^–1^ at room temperature^13^), make it a promising candidate for enhancing battery performance. However, recent studies have raised concerns about the stability of LiNbO, particularly under high-voltage conditions where it is suggested to undergo decomposition.^14,15^ This potential drawback of LiNbO highlights the importance of ongoing research and the optimization of this coating material.

It should be noted that after coating, there are two kinds of interfaces between the cathode and SEs. In this study, we focus on understanding the interface ionic transport between LiNbO and LiCoO_2_ (LCO) by comparing it with LiTaO_3_ (LiTaO), another promising coating candidate that possesses a similar structure.^16^ To obtain such insights, we employed CALYPSO methods to explicitly sample and construct the interface model.^17^ Our computational findings reveal that the Li^+^ migration through LiTaO/LCO interfaces encounters a lower energy barrier (in general less than 0.4 eV) compared to that of LiNbO/LCO (in general higher than 0.5 eV), suggesting a significant impact of interface strain on Li^+^ transport. Furthermore, we extend our analysis to evaluate how these coatings influence the degradation processes of LCO, specifically looking at oxygen at the LCO surface. This comprehensive approach not only provides insights into the ion transport mechanisms at the interface but also elucidates the role of these coatings in enhancing the overall stability and performance of the cathode material in SSBs. The insights gained from this research are expected to guide future material design and selection for more robust and efficient SSBs technologies.

## Calculation Methods

The density functional theory (DFT) method was employed within the generalized gradient approximation of the Perdew, Burke, and Ernzernhof functional as implemented in the VASP software.^18,19^ The projector-augmented wave method (PAW) was employed to represent the ionic cores by considering the following electronic states as the valence: O 2s and 2p; Li 2s and 2p; Co 3p 4s; Nb 4p 5s and 4d; Ta 5p 6s and 5d. To better treat the 3d electric orbital of Co, A “Hubbard-*U*” scheme was introduced with *U*_Co_ = 4.91 eV.^20^ A plane-wave kinetic-energy cutoff of 650 eV and a k-spacing of 0.3 were employed for the geometry optimization of LCO, LiNbO, and LiTaO unit cells. All calculations were stopped when the forces in the atoms were all below 0.01 eV Å^–1^. The interface face model selection was performed with an energy cutoff of 550 eV and halted when the forces in the atoms were all below 0.03 eV Å^–1^. The lattice parameters in this study are in good agreement with those from previous experimental work, as detailed in Table S1. Pristine interface models, comprising 500 atoms (as shown in Figure S2), represent one of the most extensive scales employed in DFT simulations to date, to the best of our knowledge.

Ab initio nudged-elastic band (NEB) calculations were conducted to calculate the Li^+^ diffusion energy barrier in bulk LCO, LiNbO, and LiTaO and in interface models. The geometry optimizations were halted when the forces on the atoms were all below 0.05 eV Å^–1^. An 1 × 1 × 1 Γ-centered k-point grid for Brillouin zone sampling and an energy cutoff of 550 eV were used.

A Li vacancy (*V*_Li_) with respect to the Li metal reservoir at site *i* is considered neutrally charged and the vacancy formation (*E*_V_) is calculated using
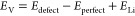
1where *E*_defect_ and *E*_perface_ represent the total energy of the structure with and without *V*_Li_*and E*_Li_ is the energy per atom of Li metal with a BCC structure, according to previous DFT results.^21^ Li energy (chemical potential μ_Li_) can be represented by −*E*_V_ (Li), which can be decomposed into:
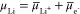
2where μ̅_Li^+^_ and μ̅_e^–^_ are the electrochemical potentials of Li^+^ and an electron, respectively. μ̅_e^–^_ is defined as the negative energy taking an electron from the highest occupied state to the Li/Li^+^ reference. μ_Li_ varies depending on its location and indicates how Li^+^ are likely to be distributed across the interface and the energy landscape they experience.^6,22^

To examine the changes in the charge distribution at the interface after coating, calculations of the charge density distribution differences (CDD) were performed. The CDD was determined using the formula in eq 5,
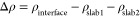
3where ρ_interface_ represents the total charge density of the interface structure, while ρ_slab1_ and ρ_slab2_ represent the isolated charge densities of the compositions of slab1 and slab2, respectively. The surface energy of the analyzed slabs, *E*_surf_, and the adhesion energy of the interface system, *E*_adh_, were calculated with the formulas:
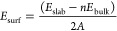
4
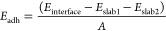
5where *E*_slab_ represents the energy of the slab after surface relaxation, *n* represents the number of formula units in the slab, *E*_bulk_ represents the bulk energy per formula unit, and A represents the surface area of the slabs. *E*_interface_, *E*_slab1_, and *E*_slab2_ indicate the total energies of the interface model and the compositions of slab1 and slab2, respectively.

## Results and Discussion

We have chosen LCO (104) and LiNb(Ta)O (11̅0) as the initial potential surface orientations for constructing the interface. (Initial bulk calculations can be found in Sec. Calculation Methods). LCO (104) stands out as one of the typical low-energy surfaces.^23^ In the case of LiNbO, prior studies have identified several possible surfaces, categorized as “X-cut,” “Y-cut,” and “Z-cut”.^24^ From these, we selected the most extensively studied surfaces, namely, (11̅0), (21̅0), and (001), and conducted surface energy calculations. LiNbO (11̅0) emerged as our target due to its notably low surface energy (see Table S2). Furthermore, LiNbO (11̅0) is recognized as one of the low-index surfaces in previous research.^22^ The interface mismatch between LCO (104) and LiNbO (11̅0) is a mere 2.3%, indicating a judicious choice for the interface model. Additionally, LiTaO (11̅0) was chosen for its similar lattice parameters to LiNbO. Our calculations demonstrate a minimal interface mismatch of 2.1% between LCO (104) and LiTaO (11̅0). We acknowledge that amorphous phases for both LiNbO and LiTaO exist. However, directly treating these amorphous structures, although ideal, requires extensive validation of the calculation results. This introduces ambiguity and increases the costs. Therefore, in this study, we focus on the fundamental and intrinsic properties of the crystalline phase of the LiNbO3 coating layer.

**Figure 1:**
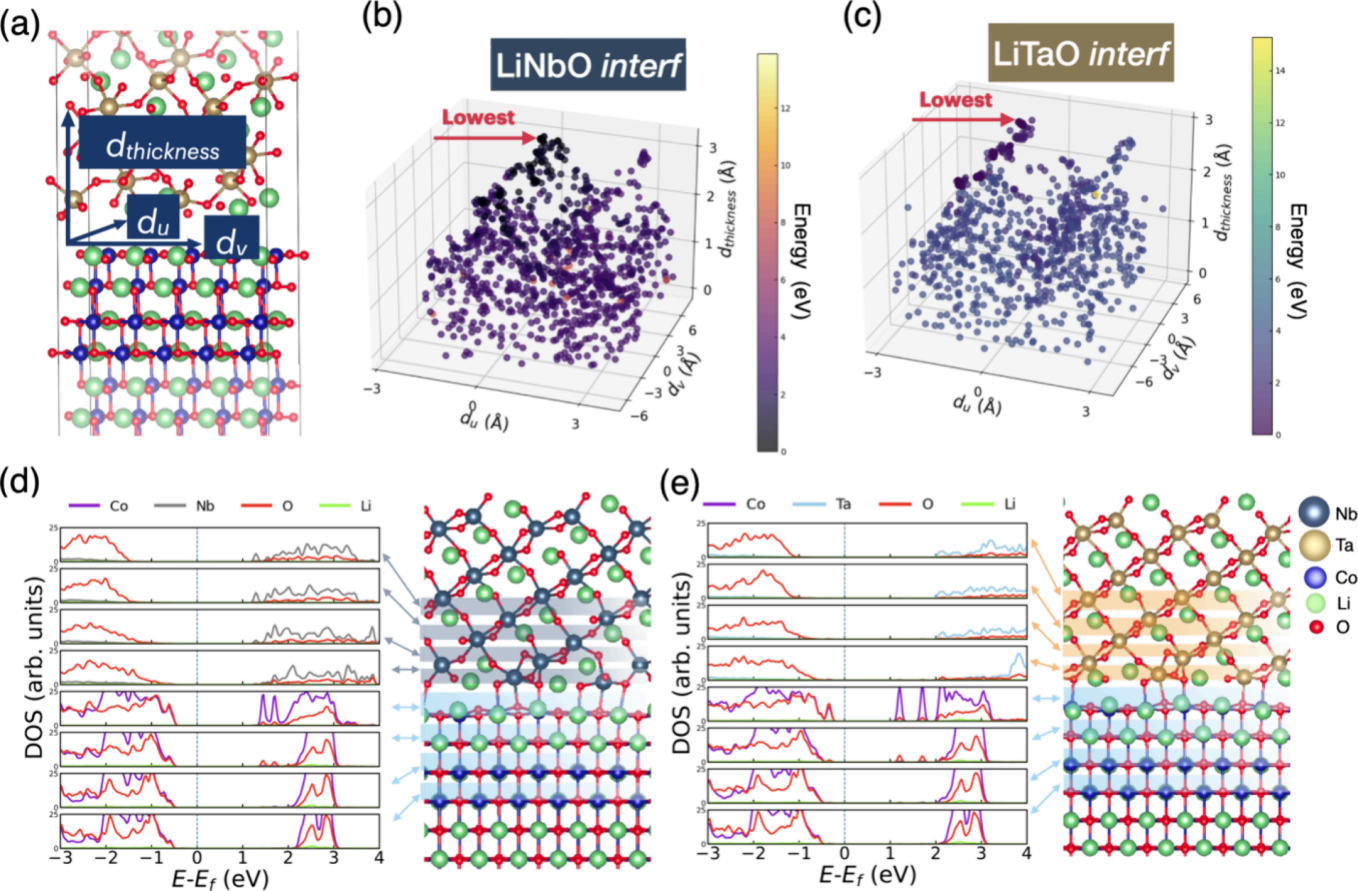
Energy distributions for (b) LiNbO *interf* and (c) LiTaO *interf* as a function of the lateral directional displacement: *d*_u_ and *d*_v_, and vertical direction: *d*_thickness_, as indicated in (a). Lowest energy configurations are indicated by red arrows, as shown in Figure S2, respectively. (d) layered Projected Density of States (PDOS) of LiNbO *interf* and (e) layered PDOS of LiTaO *interf*. Arrows point each PDOS to the corresponding layer.

To identify the most energetically favorable interface models for LiNbO (11̅0)@LCO (104) (hereafter referred to as LiNbO *interf*) and LiTaO (11̅0)@LCO (104) (hereafter referred to as LiTaO *interf*), we employed the CALYPSO methodology’s predictive scheme to generate and evaluate thousands of interface structures, focusing on energy distributions across the initial lateral (*d*_u_ and *d*_v_) and vertical (*d*_thickness_) displacements to pinpoint the most energetically favorable configurations, as shown in Figure 1a.^17,25^ The normalized energy distribution for LiNb(Ta)O *interf* is depicted as a function of displacement along three directions, as shown in Figure 1b,c, respectively. Structures exhibiting the lowest ground-state energy (indicated by red arrows in Figure 1b,c) were selected for LiNbO *interf* and LiTaO *interf*, respectively. Figure S1a,b shows the front view of both selected interface models. The calculated average interface strains are −2.3 and −2.1% for LiNbO *interf* and LiTaO *interf*, respectively, values notably smaller than the recently reported local strain (∼-3%) induced in LCO during charging.^26^ The charge density difference (CDD) plots in Figure S1c,d confirm the formation of atomic bonds critical for interface stability, with notable interactions between Nb (Ta) and O for LCO, and O with Co for LiNbO (LiTaO). Adhesion energies for LiNbO *interf* (0.79 J m^–2^) and LiTaO *interf* (0.82 J m^–2^) were calculated and indicated the formation of substantial bonds at the interface region.

To understand the coating effects of LiNbO and LiTaO, we initiated our analysis by examining the electronic structure of the interface models, which is essential for blocking electron leakage to the electrolyte. The Projected Density of States (PDOS) for LCO, LiNbO, and LiTaO bulks, as well as LiNbO and LiTaO *interf*, are shown in Figure S2a–e. To explicitly scrutinize the electronic structure variation of LCO under LiNb(Ta)O coating, we present the PDOS for each layer in interface models (Figure 1d,e). it is evident that Co and from LCO dominate the valence band maximum (VBM) in both interface cases. The valence bands in both LiNbO and LiTaO are situated in a deeper region significantly removed from the Fermi level. The band gap *E*_g_) of uncoated LCO is 2.78 eV (Figure S2a), decreasing to 1.26 eV under LiNbO coating (Figure S2d) and 1.18 eV under LiTaO coating (Figure S2e), respectively. This reduction in *E*_g_ suggests an enhanced electronic conduction at the LCO near-surface region.^27^ Meanwhile, a closer examination of the top layers in the PDOS plots in Figure 1d,e shows the reduced *E*_g_ of both LiNbO and LiTaO coatings compared with their *E*_g_ in bulk (i.e., 3.44 to 2.60 eV and 3.80 to 2.98 eV, respectively). This decrease in *E*_g_ could be ascribed to the interface dipole and strain induced by lattice mismatch. We performed additional calculations to test the *E*_g_ variations of LiNbO and LiTaO under different strains. As shown in Figure S3, tensile strain decreases the *E*_g_ for both coating candidates, consistent with the reduction of *E*_g_ at the LiNbO and LiTaO *interf*. Nevertheless, the *E*_g_ is still sufficiently large (both >2 eV) to effectively screen out electrons attempting to move toward the electrolyte. It is also noteworthy that the Conduction Band Minimum (CBM) of Nb in LiNbO is located at around 1 eV, much lower than that of Ta in LiTaO. The VBM, mainly consisting of O 2p states, in LiNbO is situated at around −1.5 eV and exhibits a more pronounced nanoscale band bending compared to the VBM in LiTaO. Such nanoscale band bending may induce possible Li deficiency and oxygen evolution from the interface region during high-voltage charging.

**Figure 2:**
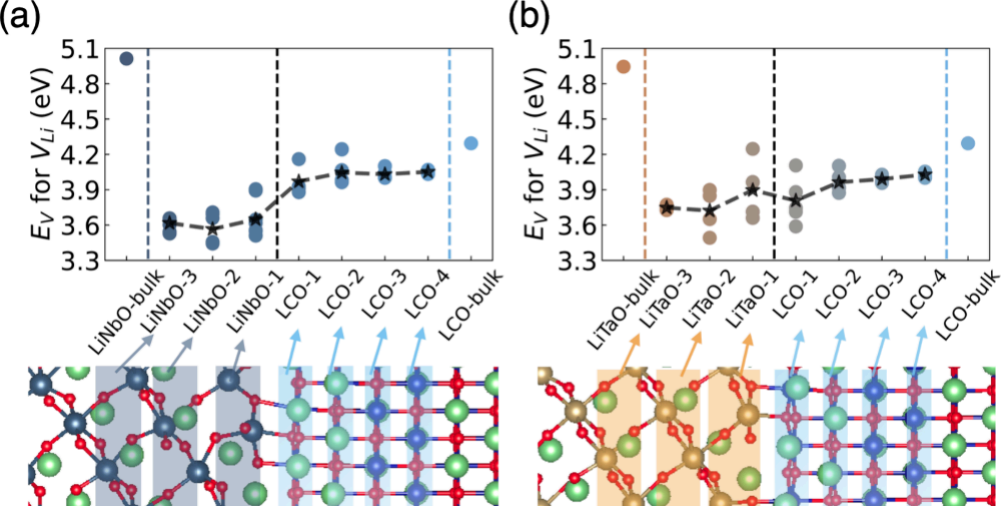
μ_Li_ as a function of layer in (a) LiNbO *interf* and (b) LiTaO *interf*. Star symbols mark the average value of μ_Li_ for each layer. Arrows point the layer names on the *x* axis to the corresponding layers. Red frames show the interface region. Star signs indicate the average μ_Li_ values of each layer. All plots share the same color code as in Figure 1.

Beyond serving as an insulator to impede electron migration, an effective coating material must also act as a buffer layer capable of lowering interfacial resistance between the cathode and electrolyte.^7,8,28^ This resistance primarily stems from the disparity in the Li chemical potential (μ_Li_) at the surfaces of cathodes and electrolytes. A significant interface resistance has been demonstrated in previous studies by comparing μ_Li_ in LCO and β-Li_3_PS_4_.^22^ To assess the impact of LiNb(Ta)O coating on LCO, we calculated the formation energy of Li vacancies as a function of layers in the interface models, as illustrated in Figure 2. Here, *E*_v_ is the negative of μ_Li_, as explained in the Calculation methods. We considered all possible Li sites in each layer, as marked by the shaded squares and arrows. The bulk phase *E*_v_ values for LiNbO, LiTaO, and LCO are 5.02, 4.96, and 4.29 eV, respectively, in good agreement with previously reported values.^22,29^

It is noticeable that the variation of μ_Li_ diminishes when Li vacancies are generated in layers more distant from the interface (illustrated by “*LiNbO-3*”, “*LiTaO-3*”, and “*LCO-4*” in Figure 2), suggesting that it approaches bulk properties with a diminishing influence from the interface formation. However, it is noteworthy that μ_Li_ (−*E*_v_) values in these layers are generally less negative than those in the bulk phase. This phenomenon can be understood through consideration of the band alignment at the interface. Upon contact with the electrolyte, electron redistribution occurs around the interface, altering the band alignment and resulting in an adiabatic potential, as documented in previous studies.^30,31^ This effect can also be observed when the electrode interfaces with the coating layer. Therefore, to rationalize our calculation of Li vacancies at the interface, we incorporated this theory by calculating the electrochemical potentials of electrons (μ̅_e^–^_) for each slab in the interface models, as shown in Tables S4 and S5. Consequently, *E*_v_ within the bulk potential can be accurately reproduced when μ̅_e^–^_ is taken into consideration. For instance, the average *μ*_*Li*_ in “*LiTaO-3*” consistently approximates −3.8 eV and μ̅_e^–^_ is approximately −1.1 eV. Utilizing eq 1, we will be able to reproduce the *E*_v_ of Li vacancy of LiTaO in its bulk value of −4.9 eV.
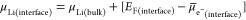
6

However, such an approximation turns out to be inadequate within the interface region, where local strain distorts the structure and new bond formations occur (see Table S3 and Figure S1c,d). Nevertheless, the μ_Li_ (−*E*_v_) calculated at the interface can be considered as the potential surface, revealing different trends in relative *E*_v_ for the two interface systems: LiTaO *interf* shows a similar average μ_Li_ at the interface (“*LiTaO-1*”, −3.9 V) compared to “*LCO 1*” (−3.8 V). Conversely, in the case of LiNbO *interf*, “*LiNbO-1*” *and* “*LCO-1*” exhibit a larger average μ_Li_ difference of approximately 0.32 V, three times higher than that for LiTaO *interf*. A larger μ_Li_ difference indicates a relatively larger Li chemical potential difference at the interface, potentially resulting in a higher energy barrier for Li^+^ migration at the interface. Such an effect has also been reported in the previous studies.^9,22^ This finding is corroborated by subsequent NEB calculations that investigate the adiabatic potential difference effect on Li^+^ migration across the interface.

**Figure 3:**
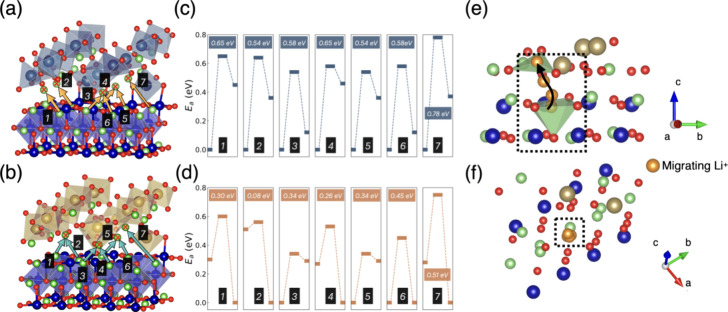
(a) and (b) Interface migration pathways for LiNbO *interf* and LiTaO *interf*, respectively. Arrows in (a) and (b) show each interface migration pathway corresponding to parts (c) and (d), respectively. The energy at initial, transitional, and final states are shown as horizontal bars in (c) and (d). Numbers in dark blue and yellow show *E*_a_ for each migration pathway. Numbers shaded black indicate the pathway index as shown in (a) and (b). Atoms with crosses indicate the final state of each pathway. (e, f) Local structures of interface migration for Li^+^ in (d) “2”. The green plane shows the oxygens coordinated with lithium Li^+^ in the initial and final state. All plots share the same color code, as shown in Figure 1.

NEB calculations were performed to thoroughly investigate and compare the Li^+^ diffusion properties across the LiNb(Ta)O *interf*, both in slabs near the interface and in bulk cases. As shown in Figure 3, seven distinct diffusion paths, labeled “1” to “7′′, have been investigated for both the LiNbO and LiTaO interfaces. Although we only consider a single vacancy hopping process, all reasonable single-vacancy migration pathways are included to cover every possible migration pathway across the interface. The interface migration distance is constrained to a maximum distance of 5 Å, a limitation imposed to mitigate the impact of voids, which can significantly increase the *E*_a_. Notably, in both interface scenarios examined, LiNbO and LiTaO, the pathway labeled “7” shows the highest *E*_*a*_. This phenomenon will be attributed to the migration distances observed, which are 4.26 Å for the LiNbO interface and 4.93 Å for the LiTaO interface.

In the case of the LiNbO interface, the calculated *E*_a_ values are predominantly above 0.5 eV, ranging from 0.54 to 0.78 eV. Conversely, the *E*_a_ values for the LiTaO interface are generally below 0.5 eV, with the exception of sample “7”. Notably, sample “2” in the LiTaO interface exhibits the lowest *E*_a_, approximately 0.1 eV. This low *E*_a_ was further investigated through an examination of the local structure. Figure 3e,f illustrates that when Li^+^ (depicted in orange) are at their final position, they coordinates with only three oxygen atoms (the green plane in Figure 3e). This is in contrast to other Li^+^ ions in either LCO or LiTaO environments, which typically coordinate with five or six oxygen atoms, leading to a lower final energy state compared to the initial one (refer to Figure 3d, “2”). Additionally, during the transition state, the Li^+^ maintains an average distance of approximately 2.2 Å from the nearest oxygen atoms, indicating a weak bonding interaction and thus contributing to the significantly lower *E*_a_. Since Li^+^ tends to migrate through the pathway with lower *E*_a_, as in LCO,^32^ the NEB calculations suggest that Li^+^ should migrate more easily to LiTaO compared with LiNbO. Moreover, NEB results agree with the chemical potential calculations at the interface, indicating the adiabatic potential landscape should be the reason for the higher *E*_a_ in LiNbO *interf.* Further, due to a similar Li^+^ potential for LiNbO and LiTaO, it is confirmed that the strain induced by lattice mismatch causes the difference in μ_Li_ at the interface region, which determines the ease of Li^+^ diffusion from the cathode to the coating layer. In addition, we further compared Li^+^ diffusion in both coating layers and LCO slabs. Related discussion can be found in Supporting Discussion (See Supporting Information).

**Figure 4:**
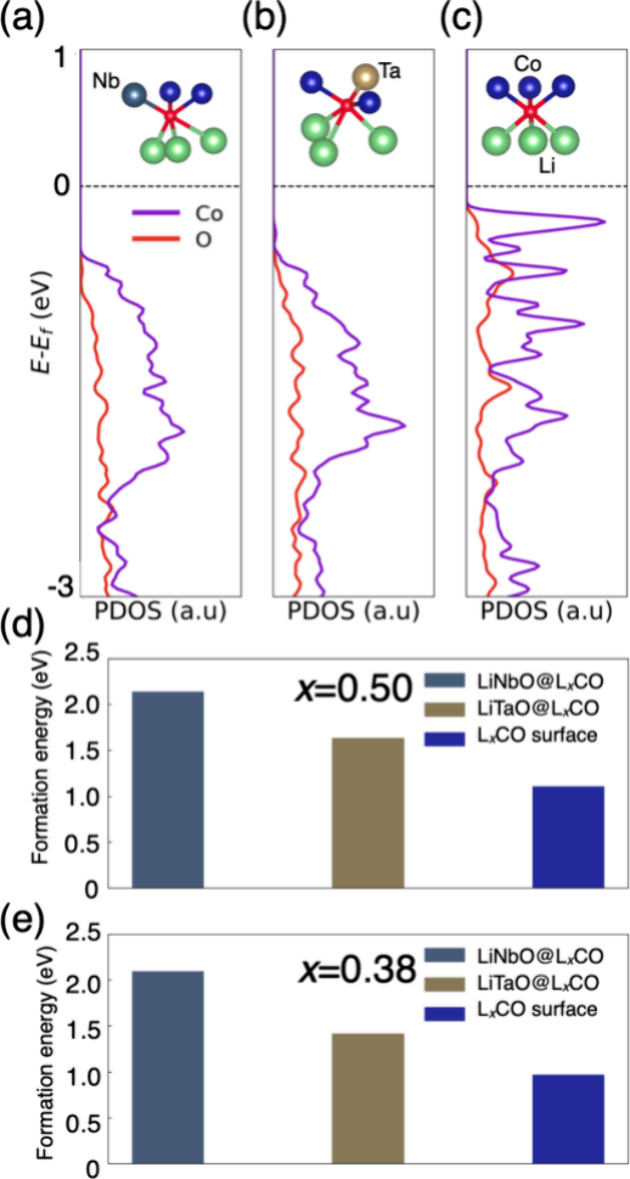
(a–c) Projected density of states (PDOS) and schematic local environment (insets) of lattice oxygen coordinated in LiNbO *interf*, LiTaO *interf,* and LCO bulk. Oxygen vacancy formation energy at the interface/surface in delithiation states: (d) *x* = 0.5 and (e) *x* = 0.38. Atom plots share the same color code as shown in Figure 1.

To further investigate the protective impact of LiNbO and LiTaO coatings on structural and thermal stability, we examined the behavior of the oxygen vacancy formation energy near the interface. First, we compared the PDOS of O 2p states in different local environments, namely, O in the LCO bulk and at the interface. Surface O, when bonded with Nb or Ta, assumes an octahedral configuration (Figure 4a, b), similar to that in the bulk LCO environment (Figure 4c). However, as shown in the PDOS in Figure 4c, O 2p has a much more intensive hybridization with Co 3d states compared with after coating (Figure 4a, b). Moreover, fewer O high-energy states are observed at the interface near the highest-occupied states (black dotted lines shown in Figure 4a–c), attributed to the Nb/Ta–O bond at the interface effectively lowering the energy of O 2p states. This indicates suppressed oxygen activity in the interface region.^33,34^

Furthermore, we evaluated the oxygen formation energy for the different highly delithiated cases. The Li concentration is set to be 50 and 38% (i.e., *x* = 0.50 and 0.38 for L_*x*_CoO_2_). These concentrations were determined based on previous experimental work, in which oxygen loss from the (104) surface was reported under delithiation conditions of *x* = 0.45.^35^ In terms of the delithiated Li configurations, we removed Li atoms from the outmost surface/interface to create a Li-poor region, as suggested by Kikkawa et al.’s observations of LCO under overcharging.^36^ There are 40 oxygen atoms in the surface/interface LCO region, and all of them are considered by removing one at a time to calculate the formation energy. At the L_*x*_CO surface, the average oxygen vacancy formation energy is ∼1.0 eV for *x* = 0.55 and 0.62 (Figure 4d), indicating that oxygen gas is prone to form at such delithiation levels. This finding aligns well with the experimental work at a similar delithiation.^35^ After coating, we observed that the formation energy increases to ∼2.0 eV under the LiNbO coating and ∼1.5 eV under the LiTaO coating (Figure 4d,e). The increased oxygen vacancy formation energies indicate the possible mitigation of oxygen release from the LCO surface. Moreover, LiNbO demonstrates a better effect in terms of increasing the oxygen formation energy. This could be due to the more localized 4d orbitals of Nb compared with the 5d orbitals of Ta, causing Nb and surface O to form stronger bonds than Ta and O. We next focused on the PDOS of O near the highest-occupied states between LiNbO and LiTaO coating, as shown in Figure S6. O 2p states are found to be pushed to a deeper region in the LiNbO coating compared with the LiTaO coating, indicating that LiNbO exhibits a better effect in suppressing the oxygen activity (evolution) at the LCO (104) surface.

## Conclusions

In conclusion, this study presents a detailed comparative analysis of LiNbO and LiTaO coatings on LCO cathodes in SSBs utilizing the DFT theory and the interface CALYPSO method. Our results highlight that LiTaO coatings demonstrate an easier Li^+^ migration across the interface, attributed to a smaller interface Li chemical potential difference due to the relatively smaller interface lattice mismatch. On the other hand, LiNbO coatings show a more pronounced effect in suppressing oxygen activity, particularly at high delithiation states. The oxygen vacancy formation energy is much increased for LiNbO compared to LiTaO. This comparative analysis between LiNbO and LiTaO coatings provides valuable insights into the design and optimization of cathode materials, emphasizing the importance of tailored surface modifications to meet specific performance criteria. Furthermore, the methodological approach adopted in this study sets a new benchmark for the investigation of interface phenomena in battery materials. The use of CALYPSO modeling combined with DFT simulations offers a comprehensive and detailed characterization at the atomic level, enabling a deeper understanding of the complex interactions and dynamics within battery systems. These findings not only confirm the advantageous properties of LiTaO and LiNbO in certain aspects but also introduce a new perspective on how these materials interact at the interface with LCO. The methodological approach adopted in this study, combining CALYPSO modeling with DFT simulations, sets a new benchmark for the investigation of interface phenomena in battery materials, enabling a more comprehensive and detailed characterization at the atomic level.

This study provides valuable insights and new findings that can guide the design and optimization of cathode materials, emphasizing the importance of tailored surface modifications to meet specific performance criteria. The comparative analysis between LiNbO and LiTaO coatings offers essential information for enhancing the overall stability and efficiency of SSB technologies.

## Supporting Information Available

Bulk and surface properties, interface sampling calculations, Li^+^ diffusion properties, and supporting discussion of Li^+^ diffusion in coating layers and LCO slabs (PDF)
