# Supplementary material for: LiNbO3 and LiTaO3 Coating Effects on the Interface of the LiCoO2 Cathode: A DFT Study of Li-Ion Transport
Source: ACS Appl Mater Interfaces. 2024 Aug 5;16(32):42093–9. doi: 10.1021/acsami.4c05737 (PMC11331435; doi:10.1021/acsami.4c05737)
Supplement: Supplementary file 1 — am4c05737_si_001.pdf [file am4c05737_si_001.pdf]

SUPPORTING INFORMATION

$LiNbO_3$ and $LiTaO_3$ coating effects on the interface of the $LiCoO_2$ cathode: A DFT study of Li-ion Transport

*Zizhen Zhou,[a,b,c] * Huu Duc Luong,[b,c] Bo Gao,[d] Toshiyuki Momma[a] and Yoshitaka Tateyama[a,b,c*]*

a, Graduate School of Advanced Science and Engineering, Waseda University, Shinjuku-ku, Tokyo 169-8555, Japan

b, Research Center for Energy and Environmental Materials (GREEN), National Institute for Materials Science (NIMS), Tsukuba, Ibaraki 305-0044, Japan

c, Laboratory for Chemistry and Life Science, Institute of Innovative Research, Tokyo Institute of Technology, Midori-ku, Yokohama 226-8501, Japan

d, College of Materials Science and Engineering, Jilin University, Changchun, Jilin 130012, China

Corresponding Author

*E-mail: zizhen_zhou@toki.waseda.jp TATEYAMA.Yoshitaka@nims.go.jp

Table S1. Comparison between lattice parameters in this work and previous experiments

		a (Å)	b (Å)	c (Å)
LCO	This work	2.79	2.79	13.95
	Ref [1]	2.82	2.82	14.05
LiNbO	This work	5.21	5.21	14.12
	Ref [2]	5.15	5.15	13.86
LiTaO	This work	5.19	5.19	13.92
	Ref [3]	5.15	5.15	13.75

Table S2. Comparison between surfaces selected from *X*-cut, *Y*-cut and *Z*-cut.

	X-cut	*Y*-cut	*Z*-cut
Selected surface	$1\bar{1}0$	$2\bar{1}0$	001
Surface energy (J m^{-2})	0.95	1.00	1.33

Table S3 Surface slab parameters of LCO(104), LiNbO($1\bar{1}0$) and LiTaO($1\bar{1}0$). |u| and |v| indicate the lengths of the surface vectors. γ represents the angle (º) between the two surface vectors. $\bar{\varepsilon}$ represents the average lattice-mismatch strain.

	$|u|$ (Å)	$|v|$ (Å)	γ (°)	$\bar{\varepsilon}$ (%)
LCO(104)	5.64	13.90	90	
LiNbO ($\mathbf{1\bar{1}0}$)	5.21	14.12	90	2.3%
LiTaO ($\mathbf{1\bar{1}0}$)	5.19	13.92	90	2.1%

Table S4 Calculation of η_{e^-} at LiTaO *interf*

$\bar{\mu}_{e^-}$	LiTaO *interf*
LCO	-0.18 eV
LiTaO	-1.08 eV

Table S5 Calculation of η_{e^-} at LiNbO *interf*

$\bar{\mu}_{e^-}$	LiNbO *interf*
LCO	-0.22 eV
LiNbO	-1.48 eV

Table S6 Comparison of E_a for Li^+ diffusion in LiTaO and LiNbO between this study and previous DFT work.

	Ref[4]	This work
LiNbO	1.29 eV	1.06 eV
LiTaO	1.23 eV	1.02 eV

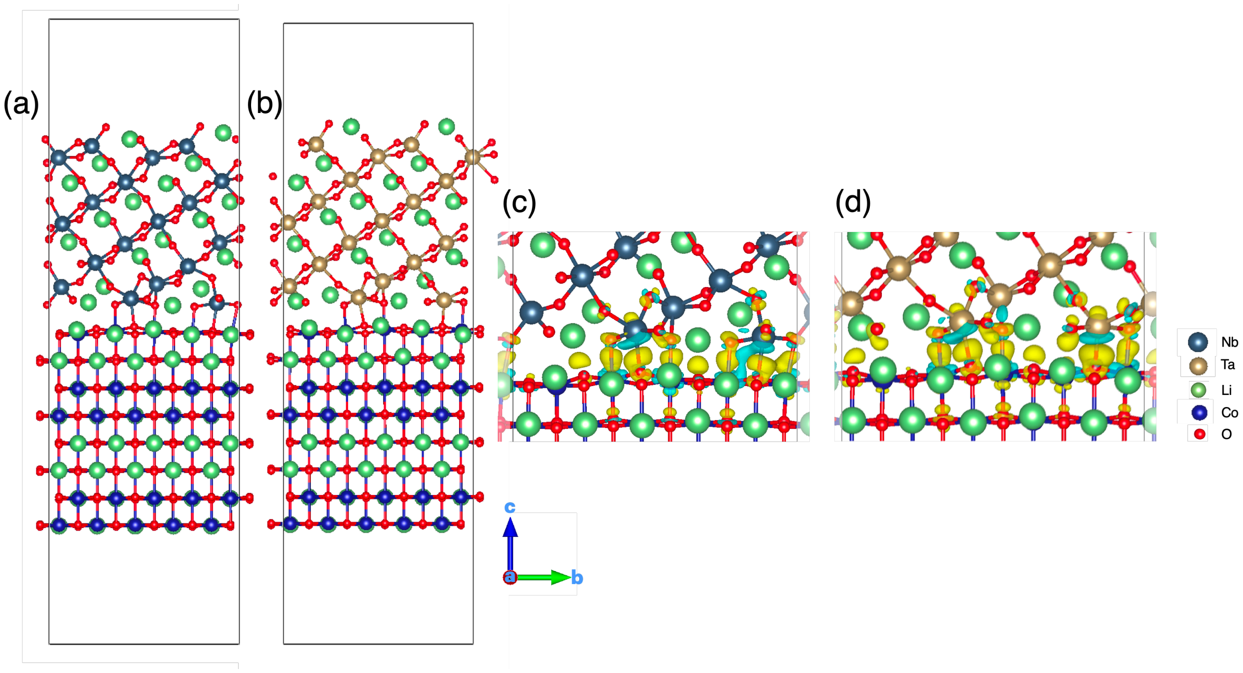

Figure S1. Schematic representations of interface models for the LiNbO coating on LCO (a) and LiTaO coating on LCO (b). Charge density difference calculated for (c) LiNbO *interf* and (d) LiTaO *interf*. Electronic charge depletion and accumulation regions are represented by cyan and yellow, respectively. The isosurface value was set to 0.01 eV Å^{-3}.

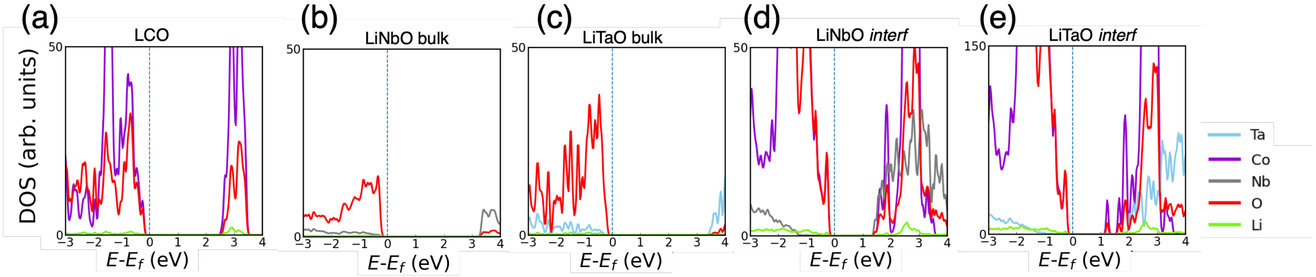

Figure S2. Projected density of states (PDOS) for (a) LCO bulk, (b) LiNbO bulk, (c) LiTaO bulk, (d) LiNbO *interf*, (e) LiTaO *interf*.

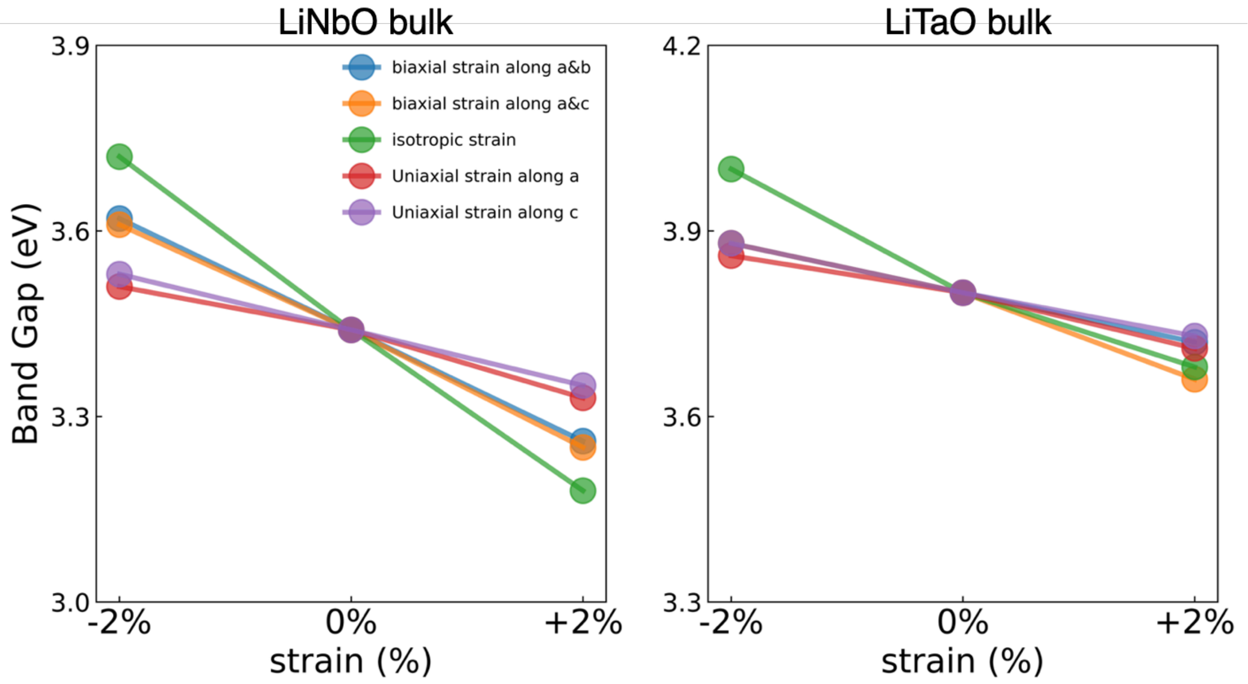

Figure S3. Band gap (E_g) as a function of strain in LiNbO and LiTaO bulk. Strain types as follows: Uniaxial strains along *a* axis, Uniaxial strains along *c* axis, biaxial strains along *a* and *b* axes, biaxial strains along *a* and *c* axes, and isotropic strains.

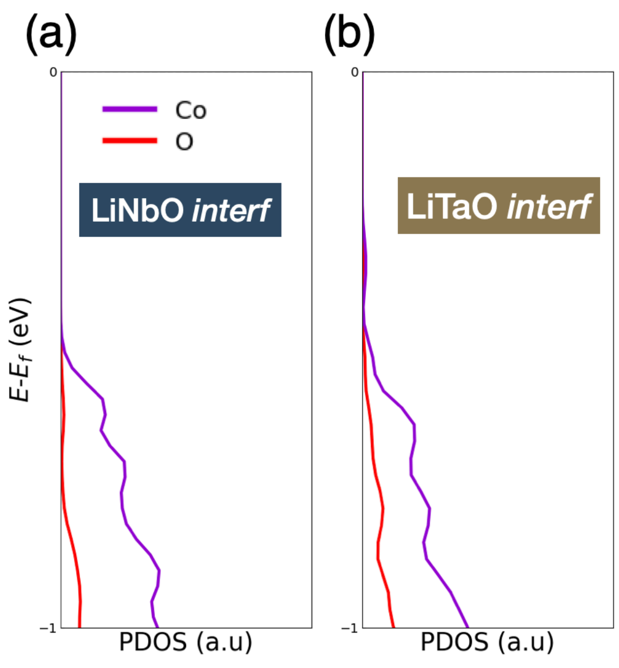

Figure S4. PDOS of surface O and Co near the highest-occupied states in (a) LiNbO *interf* and (b) LiTaO *interf*.

Supporting discussion

Diffusion properties near the interface in LCO and coating materials

Beyond the interface itself, the diffusion properties near the interface are also crucial to the electrochemical performance.[5] We therefore compared the Li^+ diffusion properties between the LCO slab and coating layers. For a clear comparison with the diffusion properties in the bulk region, Li^+ diffusion properties in bulk LCO, LiNbO and LiTaO are calculated and compared with previous work.[4] In terms of Li^+ diffusion in LCO, two types of diffusion pathways are considered: oxygen dumbbell hopping (ODH) and tetrahedral site hopping (TSH). The ODH pathway (Figure S6 (a)) represents Li^+ migration via single vacancy, whereas TSH (Figure S6 (b)) shows Li^+ migration via a tetrahedral site when there are multiple Li vacancies available.[6] E_a for ODH and TSH are 0.69 eV and 0.23 eV, respectively, in good agreement with previous calculations.[6–8] Li migration in LiNbO and LiTaO was then analyzed, considering only the nearest diffusion pathways (green images in Figure S6 (c)). Notably, the E_a for LiNbO and LiTaO measured in this work are 1.06 eV and 1.02 eV, respectively, in good agreement with previous research.[4] Figures S5 (a) and (b) demonstrate the variation in E_a within the coating layers, ranging from approximately 0.3 eV to 1.3 eV. This spread is attributed to local strain effects, which can either enhance or impede Li^+ diffusion due to changes in the local lattice space upon coating contact. For instance, within the LiNbO coating, a significant energy barrier of 1.49 eV is noted, alongside an extended Li^+ diffusion distance which increases from approximately 3.9 Å (Figure S8 (a)) in bulk LiNbO to about 4.5 Å. Conversely, a substantially lower E_a of ~0.32 eV is observed, with a markedly shortened Li^+ diffusion distance, decreasing from ~3.9 Å to ~2.7 Å. These findings are consistent with our supplementary NEB calculations, wherein we applied tensile and compressive strain to alter the Li^+ diffusion distance in bulk LiNbO, as depicted in Figure S7. Additionally, certain lengthy diffusion pathways with low E_a were identified, as illustrated in Figure S5 (a) and (b). We analyzed the Li^+ diffusion pathway in bulk LiNbO and found a tendency for Li^+ to bond with O (Li labeled '3' and O indicated by a cross in Figure S8 (a)), with the breaking of this bond leading to the highest energy barrier, as seen in Figure S8 (b). It is posited that longer distances between Li and this specific O (originally 0.96 Å from Figure S8 (a)) correlate with weaker bonding interaction, consequently leading to a lower E_a value. Upon examining pathways with extended diffusion distances of yet lower E_a, it was discovered that this atomic separation increased to over 1.5 Å, elucidating the observed lower E_a values.

Regarding Li^+ diffusion on the LCO side, Figures S7 (c) and (d) illustrate that the E_a along the ODH pathway decreases from the middle of the slab (reflecting bulk properties) toward the interface. For example, in LiNbO *interf*, E_a decreases from 0.63 eV to 0.47 eV. Interestingly, Li^+ diffusion along the TSH pathway does not change much, maintaining at around 0.17 eV. Such negligible variation along the TSH pathway under the strain effect has been observed in previous DFT calculations of Li^+ diffusion.[9] Consequently, the presence of the coating is expected to facilitate Li^+ diffusion in LCO due to the lower E_a along the ODH pathway and within the coating layer itself.

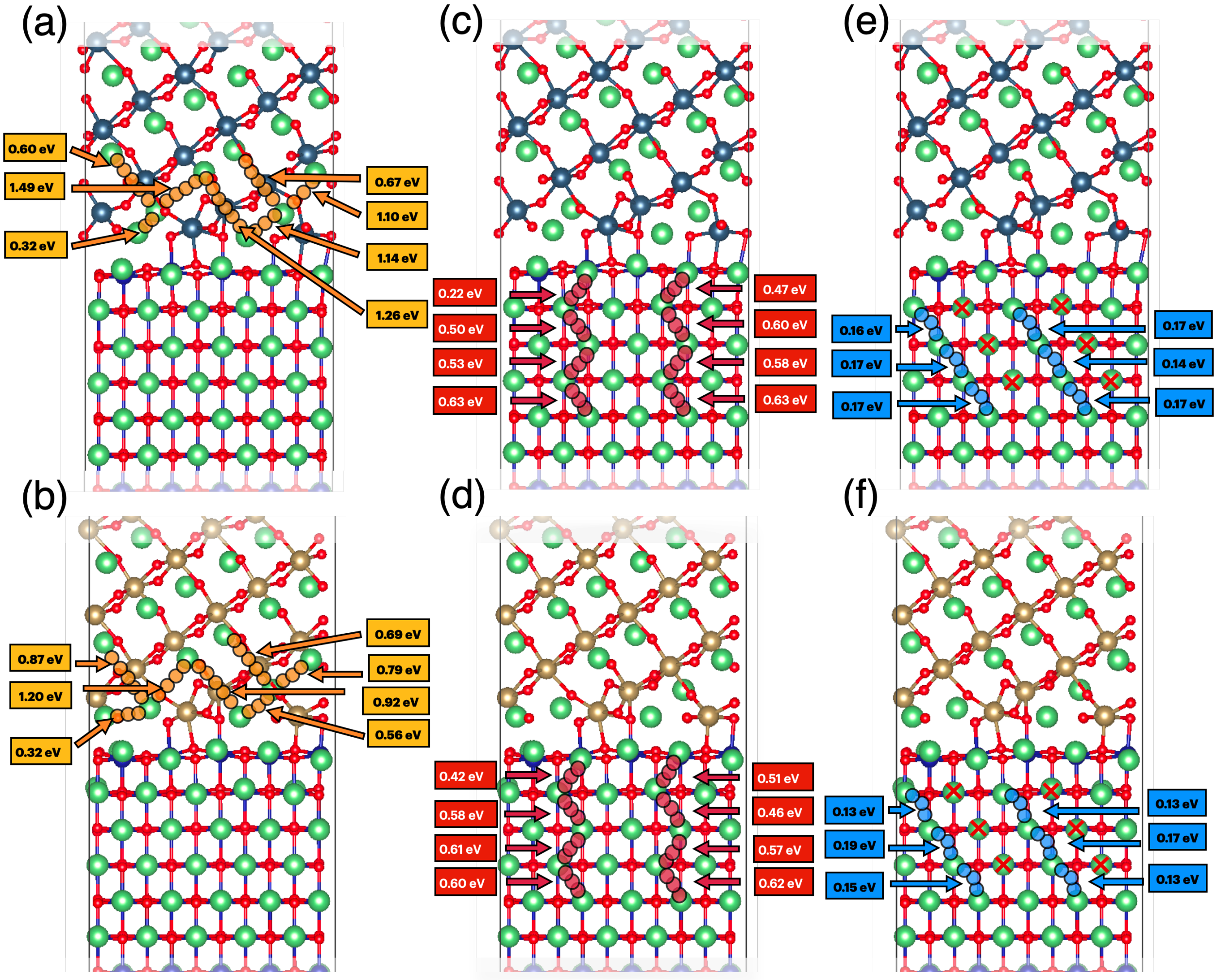

Figure S5. Li$^+$ diffusion pathway (a) in LiTaO coating layers, (b) LiNbO coating layers; along the ODH pathway in LCO in (c) LiNbO *interf* and (d) LiTaO *interf*; along TSH in LCO in (e) LiNbO *interf* and (f) LiNbO *interf*. Yellow, red and blue circles show the diffusion images of Li$^+$. Red crosses mark the additional Li vacancy for the TSH diffusion pathway. All plots share the same color code as Figure 1.

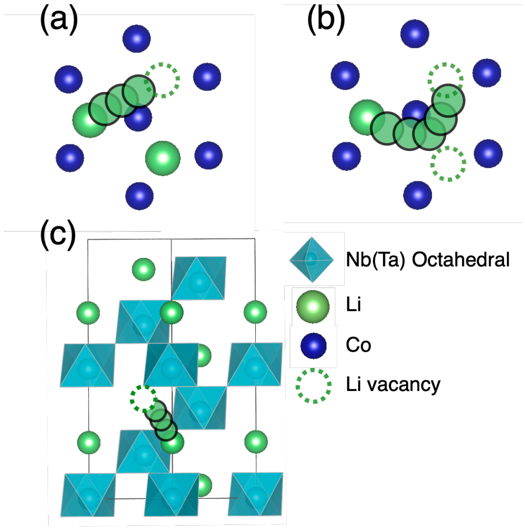

Figure S6. Diffusion pathway for (a) ODH and (b) TSH in LCO. (c) Green circles show the first nearest neighbor (NN).

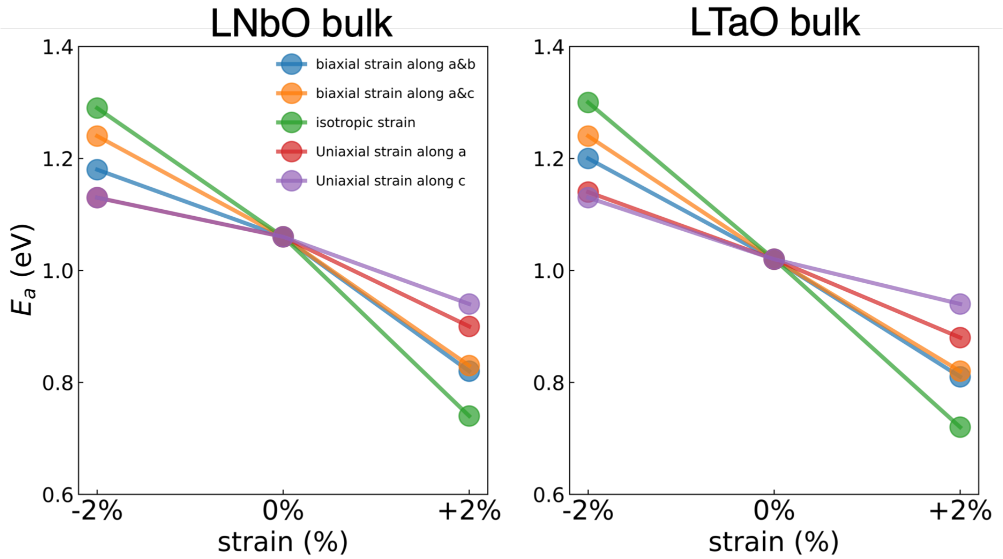

Figure S7. Migration energy barrier (E_a) as a function of strain in LiNbO and LiTaO bulk. Strain types include uniaxial strains along *a* axis, uniaxial strains along *c* axis, biaxial strains along *a* and *b* axes, biaxial strains along *a* and *c* axes, and isotropic strains.

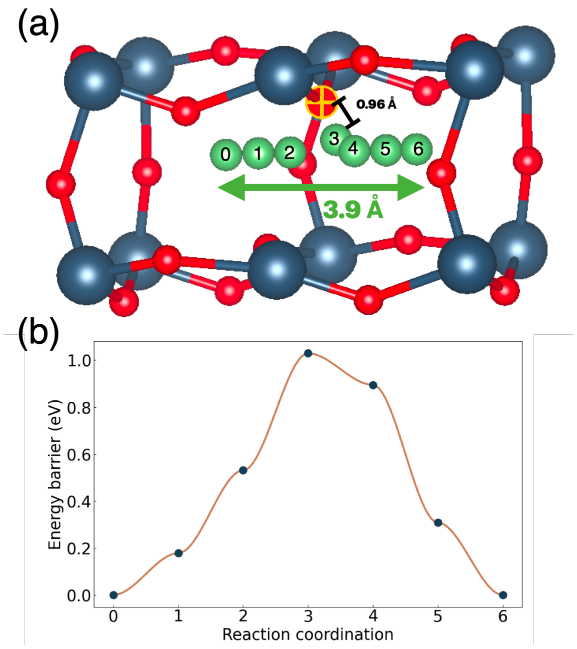

Figure S8. (a) Migration pathway of Li^+ in LiNbO bulk. Diffusion distance is indicated in green. Li^+ and O bond distance is indicated in red. Numbers indicate the reaction coordination in (b). A cross marks the closed oxygen atom with the diffusing Li^+.

Reference

(1) Van Elp, J.; Wieland, J. L.; Eskes, H.; Kuiper, P.; Sawatzky, G. A.; De Groot, F. M. F.; Turner, T. S. Electronic Structure of CoO, Li-Doped CoO, and $LiCoO_2$. *Phys Rev B* **1991**, *44* (12), 6090–6103. https://doi.org/10.1103/PhysRevB.44.6090.

(2) Abrahams, S. C.; Marsh, P. Defect Structure Dependence on Composition in Lithium Niobate. *Acta Crystallogr B* **1986**, *42* (1), 61–68. https://doi.org/10.1107/S0108768186098567.

(3) Muthurajan, H.; Kumar, H. H.; Natarajan, N.; Ravi, V. A Novel Technique to Prepare $LiTaO_3$ at Low Temperature. *Ceram Int* **2008**, *34* (3), 669–670.

(4) Kofahl, C.; Dörrer, L.; Muscutt, B.; Sanna, S.; Hurskyy, S.; Yakhnevych, U.; Suhak, Y.; Fritze, H.; Ganschow, S.; Schmidt, H. Li Self-Diffusion and Ion Conductivity in

Congruent $LiNbO_3$ and $LiTaO_3$ Single Crystals. *Phys Rev Mater* **2023**, *7* (3). https://doi.org/10.1103/PhysRevMaterials.7.033403.

(5) Zhou, Z.; Chu, D.; Gao, B.; Momma, T.; Tateyama, Y.; Cazorla, C. Tuning the Electronic, Ion Transport, and Stability Properties of Li-Rich Manganese-Based Oxide Materials with Oxide Perovskite Coatings: A First-Principles Computational Study. *ACS Applied Materials & Interfaces* **2022**, *14* (32), 37009–37018. https://doi.org/10.1021/acsami.2c07560.

(6) Van der Ven, A.; Ceder, G. Lithium Diffusion in Layered Li_xCoO_2. *Electrochemical and Solid State Letters* **2000**, *3* (7), 301. https://doi.org/https://dx.doi.org/10.1149/1.1391130.

(7) Moradabadi, A.; Kaghazchi, P.; Rohrer, J.; Albe, K. Influence of Elastic Strain on the Thermodynamics and Kinetics of Lithium Vacancy in Bulk $LiCoO_2$. *Phys Rev Mater* **2018**, *2* (1), 015402. https://doi.org/10.1103/PhysRevMaterials.2.015402.

(8) Luong, H. D.; Xu, C.; Jalem, R.; Tateyama, Y. Evaluation of Battery Positive-Electrode Performance with Simultaneous Ab-Initio Calculations of Both Electronic and Ionic Conductivities. *J Power Sources* **2023**, *569*. https://doi.org/10.1016/j.jpowsour.2023.232969.

(9) Zhou, Z.; Cazorla, C.; Gao, B.; Luong, H. D.; Momma, T.; Tateyama, Y. First-Principles Study on the Interplay of Strain and State-of-Charge with Li-Ion Diffusion in the Battery Cathode Material $LiCoO_2$. *ACS Appl Mater Interfaces* **2023**. https://doi.org/10.1021/acsami.3c14444.
